# The μDBS: Multiresolution, Directional Deep Brain Stimulation for Improved Targeting of Small Diameter Fibers

**DOI:** 10.3389/fnins.2019.01152

**Published:** 2019-10-29

**Authors:** Daria Nesterovich Anderson, Connor Anderson, Nikhita Lanka, Rohit Sharma, Christopher R. Butson, Brian W. Baker, Alan D. Dorval

**Affiliations:** ^1^Department of Biomedical Engineering, College of Engineering, University of Utah, Salt Lake City, UT, United States; ^2^Department of Neurosurgery, School of Medicine, University of Utah, Salt Lake City, UT, United States; ^3^Scientific Computing and Imaging (SCI) Institute, University of Utah, Salt Lake City, UT, United States; ^4^Department of Electrical and Computer Engineering, University of Utah, Salt Lake City, UT, United States; ^5^Department of Neurology, School of Medicine, University of Utah, Salt Lake City, UT, United States; ^6^Department of Psychiatry, School of Medicine, University of Utah, Salt Lake City, UT, United States; ^7^Utah Nanofab, University of Utah, Salt Lake City, UT, United States

**Keywords:** deep brain stimulation, directional electrodes, electrode fabrication, computational modeling, neural targeting

## Abstract

Directional deep brain stimulation (DBS) leads have recently been approved and used in patients, and growing evidence suggests that directional contacts can increase the therapeutic window by redirecting stimulation to the target region while avoiding side-effect-inducing regions. We outline the design, fabrication, and testing of a novel directional DBS lead, the μDBS, which utilizes microscale contacts to increase the spatial resolution of stimulation steering and improve the selectivity in targeting small diameter fibers. We outline the steps of fabrication of the μDBS, from an integrated circuit design to post-processing and validation testing. We tested the onboard digital circuitry for programming fidelity, characterized impedance for a variety of electrode sizes, and demonstrated functionality in a saline bath. In a computational experiment, we determined that reduced electrode sizes focus the stimulation effect on small, nearby fibers. Smaller electrode sizes allow for a relative decrease in small-diameter axon thresholds compared to thresholds of large-diameter fibers, demonstrating a focusing of the stimulation effect within small, and possibly therapeutic, fibers. This principle of selectivity could be useful in further widening the window of therapy. The μDBS offers a unique, multiresolution design in which any combination of microscale contacts can be used together to function as electrodes of various shapes and sizes. Multiscale electrodes could be useful in selective neural targeting for established neurological targets and in exploring novel treatment targets for new neurological indications.

## Introduction

Deep brain stimulation (DBS) is a widely accepted therapy for several movement disorders and an emerging therapy for psychiatric disorders and additional movement disorders. From its first FDA approval for essential tremor in 1997, the physical design of DBS leads has remained largely unchanged (Eisinger et al., 2019). A cylindrical shaft with four cylindrical electrode contacts defines the classic lead design. In this manuscript, we present a novel neurostimulation device that assembles multiresolution electrodes from microscale contacts to enable fine control of the stimulation volume and an improved capability to target small-diameter fibers.

In recent years, the FDA has approved more lead designs from major neuromodulation companies, however, these leads differ minimally from the classic quadripolar lead design. Moderate advances to the classic lead design involve contacts capable of directionally focusing stimulation, typically by having two of the four contacts subdivided into three smaller contacts each. These smaller, directional contacts allow for directional steering of the activation field to, ideally, activate the target structure while avoiding side-effect-inducing regions that might reduce the window of therapy.

Directional stimulation has already been clinically demonstrated to widen the therapeutic window by steering stimulation away from regions that may be responsible for inducing side effects (Steigerwald et al., 2016; Dembek et al., 2017). Other experimental lead designs have further subdivided contacts to allow for finer directional control and have shown promising results at widening the therapeutic window (Contarino et al., 2014; Pollo et al., 2014). However, the fundamental limitation in repeatedly subdividing contacts is enclosing enough wires for each contact within the lead shaft without increasing the width of the lead. With the technology available today, the ability to increase the number of stimulation electrodes will remain limited without further advances in lead technology.

We propose a novel directional DBS device, the μDBS, with hundreds of individually controllable contacts capable of stimulation and recording. Using onboard circuitry, the lead can stimulate using any combination of contacts at 7 independent voltage states with only 12 input wires. Multiresolution electrode sizes and complex monopolar and bipolar configurations are achievable by grouping contacts according to the desired stimulation bus lines. Such flexibility enables electrodes to scale in size from the ∼6.0 mm^2^ of the classic clinical electrode down to the ∼0.02 mm^2^ of a single μDBS contact. Here, we outline the design steps, fabrication, and bench testing of this novel, multi-resolution DBS device.

We aim to create a DBS device with the capability of stimulating through variously sized electrodes composed of contacts that are orders of magnitude smaller than those currently available in the clinic. In many instances, the side-effect-inducing regions comprise larger fibers than those most associated with therapeutic benefit (Lang et al., 1999; Chaturvedi et al., 2010). In this paper, we expand upon our recent computational work that smaller electrodes more efficiently activate small diameter fibers over large diameter fibers (Anderson et al., 2019). Smaller contacts may also widen the therapeutic window by preferentially activating smaller, therapeutic fibers over larger, side effect-inducing fibers. The present work supports that multiresolution stimulation devices can substantially improve neuromodulation efficiency and selectivity, and demonstrates the practicality of building one such device, the μDBS, as part of the next generation of neuromodulation therapy.

## Materials and Methods

**FIGURE 1:**
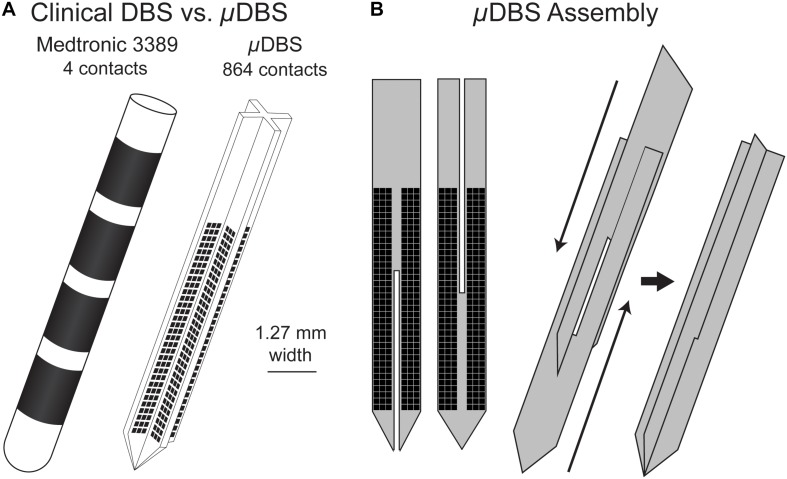
**(A)** Clinical deep brain stimulation electrode (left) with four contacts, and the μDBS (right) with hundreds of contacts. **(B)** The μDBS electrode is assembled from four total flat chips, with two flat chips paired back to back. The paired chips are assembled together to form a “+” shape when viewed from above.

We designed a novel DBS lead, the μDBS, as a microelectrode array appropriate for deep brain stimulation. This new lead has a similar scale to those used clinically, but comprises 864 microscale contacts instead of 4 large contacts. This design expands upon our first iteration of the μDBS (Willsie and Dorval, 2015b) with an improved fabrication process, slightly larger stimulation contacts, and increased stimulation flexibility via the incorporation of seven (cf. three) stimulation bus lines. The novel lead is fabricated using silicon wafer-based technology, and its on-board digital circuitry allows for full control to open or close any combination of the 864 contacts using only 12 input wires. The small contact size on the μDBS—0.0225 mm^2^ compared to the 6 mm^2^ for the clinical electrode (Lanotte et al., 2002)—allows for the μDBS to have 864 total contacts and still match the overall size of clinically available leads, having a width of 1.27 mm (Figure 1A). A complete lead is assembled from four silicon chips consisting of 216 contacts each: two pairs of flat chips are assembled front-to-back, and the two pairs are slid past each other to form a plus-shaped cross section (Figure 1B). Through our redesign of DBS lead technology, the μDBS is the first DBS lead of similar size to the clinical leads capable of stimulating through multiresolution electrodes made up of hundreds of microscale contacts.

### Design and Fabrication

In order to achieve hundreds of individually controllable contacts, the μDBS must have on-board digital circuitry, unlike modern DBS leads that have a single wire to power each conductive contact. Each μDBS contact is programmable to eight possible states using three-bit digital logic (2^3^ = 8). Seven of the eight states tie the contact to bus lines that can be used to stimulate or record, and the last state is reserved as an unconnected, floating state. Each bus line active state is independent from the others, which allows for flexibility in stimulation, frequency, pulse width, and waveform shape for each electrode used on the lead. Having these multiple independent sources allows for greater spatial and temporal flexibility in stimulation shaping since electrodes could take on various shapes, be used in complex multipolar and bipolar configurations, and deliver unique stimulation waveforms.

**FIGURE 2:**
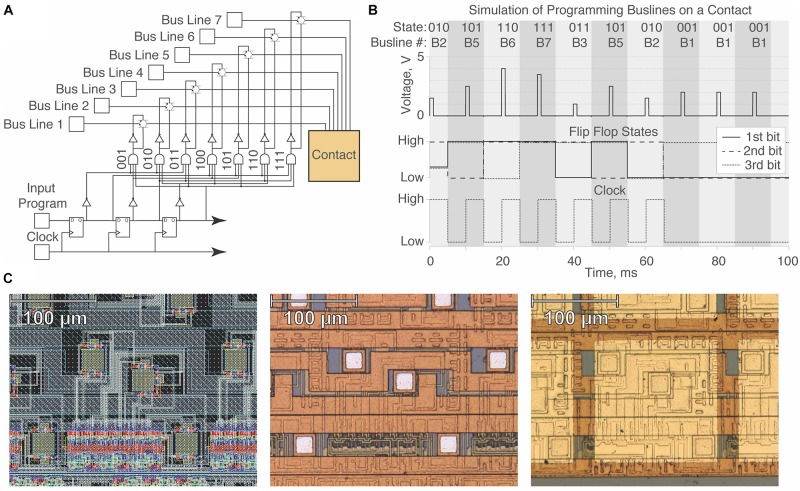
Design and simulation of a single contact unit on the μDBS. **(A)** Single contact circuit diagram with three-bit digital logic for the gating of seven bus lines. **(B)** Simulation demonstrating programming of different bus lines on a single contact in Cadence ADE XL. With the example bit stream, 011101000, we demonstrate programming the flip flop states at the falling phase of the clock signal. **(C)** Integrated circuit layout design of a single contact used in the simulation (left), post-fabrication view of the VLSI design (middle), and view of contact after gold application in post-processing (right). Note that for any moment in time, at most 1 of the 7 bus lines can be connected to a contact (large, bright gold square at right) through one of its three subcontact conduits (small, dull white squares shown in the middle panel).

Programming the device requires transmitting a serial program of three-bit “words,” where each word determines the bus line to which the contact will be tied. Each contact stores three bits of information across a shift register (serial cascade of three flip flops) and advances each bit during the falling phase of a clock signal until all contacts have been programmed to the intended state (Figure 2A). We tested whether contact states could be theoretically programmed using three-bit digital logic through the simulation of a single contact circuit prior to fabrication by X-FAB (Figure 2B). Given the presence of onboard circuitry and the serial nature of the circuit design, all contacts are controllable with a minimal number of wires using five inputs (input program, clock, power, ground, power switch) and up to seven different bus line inputs.

A layout design was made in Cadence Virtuoso using the XC06 (0.6 μm) technology package from X-FAB foundry (X-FAB, Erfurt, Germany). Circuitry for a single contact unit can be found in the left panel of Figure 2C, and the VLSI design was validated using the Cadence ADE XL package. Images of the single contact post-fabrication and post-processing can be found in Figure 2C, in the middle and right panels, respectively. Circuitry associated with one contact resides within a 165 μm × 165 μm patch, enabling a total contact size of 150 μm × 150 μm with 15 μm spacing between contacts. The primary fabrication of the design was performed by X-FAB, and post-processing fabrication work was performed in the Utah Nanofab Cleanroom at the University of Utah.

**FIGURE 3:**
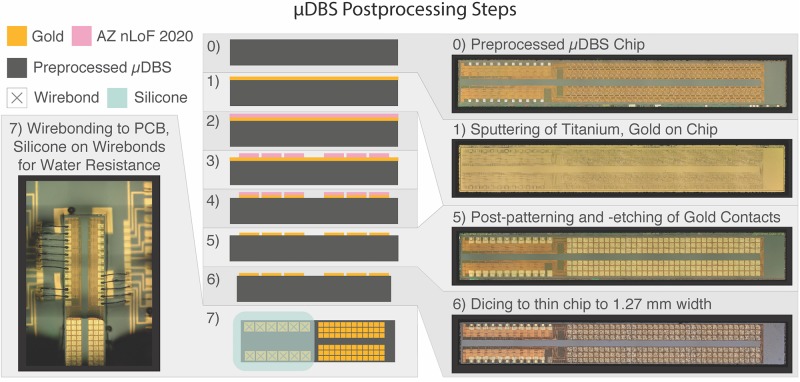
Design architecture for μDBS post-processing. Fabricated chips (0) undergo gold deposition (1) and are covered with AZ nLoF 2020 negative photoresist (2). Photoresist is exposed to UV light according to the desired contact layout through a photolithography mask and regions of photoresist not exposed to light are removed (3). Gold and titanium layers are etched away from regions not covered by photoresist to define the gold contacts (4). Remaining photoresist is washed off (5) and the chips are diced to the appropriate size (6). Connection pads are wirebonded to test PCBs (7) to enable device programming and functionality testing. Silicone was used to insulate non-contact regions from water exposure during the validation experiments.

The foundry-fabricated chips include three small subcontact pads per contact unit that underwent further processing to be linked into a single contact (see Figure 2C). Additionally, the unprocessed contact pads used Al contacts (0.5% Cu) which are not biocompatible. Chips from the foundry were sputtered with a titanium adhesion layer (∼30 nm), followed by ∼270 nm of gold, which is non-toxic and non-reactive to tissue (Merrill et al., 2005). Afterward, the chips underwent photolithography and patterning of negative photoresist (AZ nLoF 2020) in the shape of the desired contact size, at 150 μm × 150 μm (Figure 3). We exposed the patterned chips to a gold etch (8% I_2_, 21% KI, 71% DI) and a titanium etch (20:1:1 DI:HF:H_2_O_2_) to clear the titanium/gold layer from non-contact areas. Afterward, we diced the test structures placed during fabrication along the edge of the chip to match the width of the clinical electrode sizing of 1.27 mm using a diamond blade saw 70 μm in width. Following the post-processing and cutting of the device, we mounted and wirebonded the chips using aluminum wire onto a custom-printed PCB to enable μDBS programming through a computer. An interface piece of silicon with gold traces was used to facilitate wirebonding from the μDBS chip to the PCB.

The design and fabrication steps discussed in this section outline novel technology necessary to build DBS leads capable of multiresolution electrode sizes for unprecedented stimulation flexibility. The onboard circuitry and three-bit programming logic enables each contact to be individually controllable, and full functionality of the device can be achieved through only twelve wires. In the following section, we demonstrate functionality of the μDBS design through a series of programming, impedance, and stimulation bench tests.

### Validation

**FIGURE 4:**
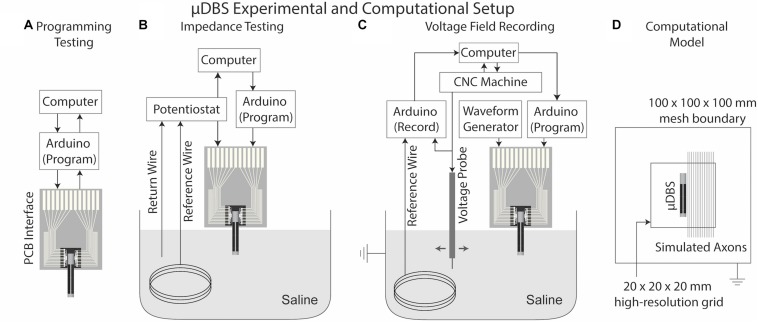
μDBS experimental and computational setup. **(A)** Experimental setup for programming requires only input/output information from the μDBS chip interfaced with the Arduino and computer. **(B)** Impedance testing requires a potentiostat connected to one bus line of the μDBS, a Pt counter wire, and an Ag/AgCl reference wire in a saline bath. **(C)** Bath testing uses a CNC machine to move a voltage probe in the saline bath around the μDBS. Voltage recordings run through a peak detection circuit and on to the Arduino for recording. **(D)** A lead-in-the-box model was used to simulate the voltage spread; multicompartment models were used to measure the effects of contact size on activation for 2.0, 5.7, and 10.0 μm diameter axons.

We assessed our ability to program the μDBS through a series of bench tests. In the scope of this section, we examined the functionality of the μDBS by fabricating and testing single flat chips that have 216 contacts each. The first instance of testing determined the accuracy of programming an intended contact configuration (Figure 4A). We measured the success rate of programming each contact state during the falling phase of the clock cycle. A numerically randomized series of 648 binary numbers (i.e., ones and zeros) was generated to program three bits on each of the 216 contacts using an Arduino programming setup, repeated five times per chip at six different clock speeds. Programming errors were quantified on a total of ten chips by comparing the fidelity of the bit program after it had passed through the chip to the series of bits that were programmed into the chip. The Arduino setup — essentially serving as the analog to an implanted pulse generator — was used to simultaneously power the device, generate the randomized programming file used to set contact states, and verify programming fidelity of the μDBS.

Additionally, we measured changes in impedances in a saline bath based on the number of contacts recruited. Increasing the number of contacts recruited to a single bus line increases the surface area of the effective electrode. The total electrode impedance was expected to vary with approximate inverse proportionality to the electrode surface area. To test proper contact recruitment, we prepared a saline solution (0.1 w/v% NaCl)—with approximately the conductivity of brain tissue (0.2 S/m)—to simulate the expected impedance of the electrode when exposed to a biological environment. Impedances were measured on a commercial electrochemical test system (Gamry Instruments PC4 Potentiostat, Warminster, PA, United States) across a Ag/AgCl reference electrode, a Pt wire counter electrode, and active contacts of the μDBS as the working electrode (Figure 4B). Impedances were quantified over a frequency range of 10 Hz to 10 kHz with a sinusoidal input voltage of 10 mV. The number of active contacts constituting the active μDBS electrode varied from 1 to 108, and each configuration was repeated three times for each of three chips.

Finally, we experimentally measured the stimulation field produced by the μDBS for two electrode configurations using a Ag/AgCl voltage probe manipulated by a computer numerical control (CNC) machine in a saline bath that matched the conductance of neural tissue (Figure 4C). The CNC machine moved the probe at a 0.5 mm resolution in a 20 mm × 10 mm grid in front of the μDBS chip in the saline solution, and voltage profiles were recorded by a separate recording Arduino setup linked to the CNC machine. The purpose of this experiment was to verify that stimulation can be done with simultaneous bus lines at different settings. The chip was functionally split in two, with 48 contacts on one half of the chip tied to bus line A and another 48 contacts on the other half tied to bus line B. For one condition, bus line A was 1.5 V and bus line B was 3.0 V with 100 μs, charge-balanced pulses; for a second condition, the bus lines were swapped. Stimulation profiles were collected for one chip in three trials for both conditions to evaluate whether the measured fields generated by the contacts were consistent with their bus line assignments.

### Computational Model

To support the need for a multiresolution device with contacts as small as 150 μm × 150 μm, we simulated computational axon models to assess the influence of varying electrode sizes on neuronal activity. Each vertical column on the μDBS comprises 36 contacts. We ran bioelectric field solutions in SCIRun 4.7 (Scientific Computing and Imaging (SCI), Institute, University of Utah, Salt Lake City, UT, United States) for 1–36 adjacent contacts within a column set to −1 V each with the surrounding box set to 0 V. These configurations resulted in electrode sizes from 150 μm × 150 μm to 150 μm × 6 mm. We implemented a high-resolution submesh with 0.1 mm spacing around the electrode, as we have described previously (Anderson et al., 2018b, 2019), and we set tissue conductivity to 0.2 S/m (Figure 4D). Non-contact regions of the μDBS were modeled as ideal insulators, and the contacts were modeled as ideal conductors. Axons of various diameters — 2.0, 5.7, and 10.0 μm — were placed parallel to the lead in 0.1 mm increments, from 0.1 to 10 mm away. The vertical axonal orientation was chosen to match that of the active electrode on the μDBS, to explore the effects of electrode size on neuron activation patterns. Simulations were run in NEURON 7.4 using the MRG neuron model (McIntyre et al., 2002), on which modeled extracellular potentials were mapped directly onto node, paranode, and internode segments. Thresholds were identified for a 90 μs charge-balanced pulse at ∼0.01 V resolution to quantify the role of electrode size on neural selectivity as a function of fiber diameter.

## Results

We conducted bench testing to evaluate the functionality of the fabricated and post-processed μDBS chips. We determined whether chips met our design specifications, as well as whether contacts could be programmed and recruited into larger electrodes through programming testing and testing in a saline bath.

### Design Verification

A series of sixteen chips were slated for post-processing and subsequent testing. Of those, six were irrevocably damaged, primarily at the wirebonding post-processing step. Table 1 summarizes the design specifications and results retrieved for the ten surviving chips. Final chip widths were ∼1.29 mm, and well within 5% tolerance of 1.27 mm design specification used to match the clinical lead. Most devices (7/10) met our form-factor and bus line acceptance criteria. In the other devices (3/10), wirebonding failed to connect all seven bus lines; but note that these chips could still be used with somewhat reduced flexibility through their 4–6 functioning bus lines. We attempted gold contact patterning on eight of the ten chips, and they all met acceptance criteria. On average, the gold contact widths and heights measured slightly smaller than designed, possibly because of chemical undercutting from the gold and titanium etch during photolithography. To accommodate this undercut in future iterations, we will simply enlarge the contacts in the photolithography mask. In summary, gold contact patterning was universally successful, and the majority of chips met all acceptance criteria; for chips that did not meet acceptance criteria, wirebonding was the most common failure point.

**TABLE 1:** Design verification to determine whether devices met design specifications.

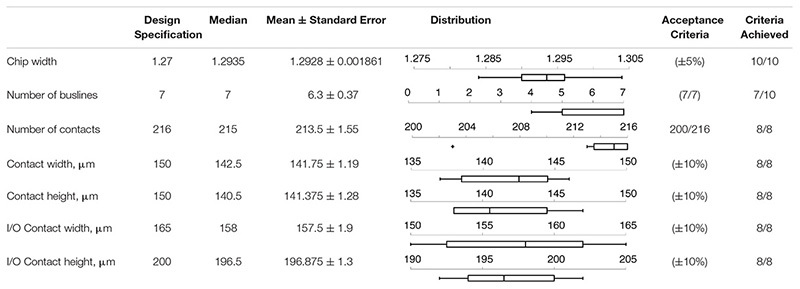

### Programming Testing

**FIGURE 5:**
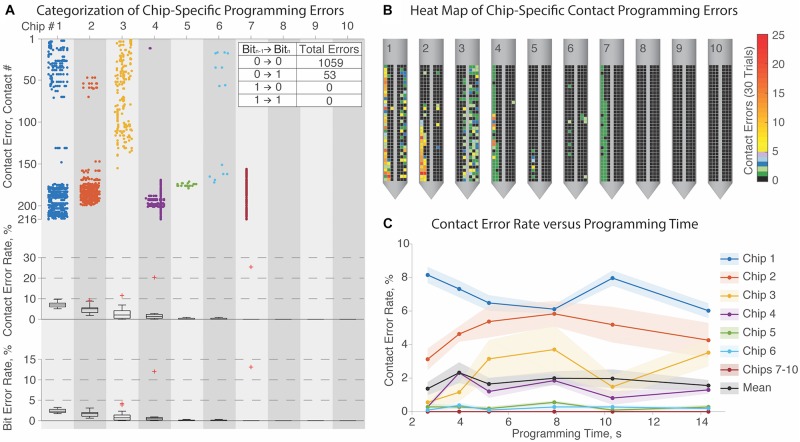
Programming validation of μDBS chips. **(A)** Characterization of contact errors for 10 μDBS chips across 30 trials each, with incorrectly programmed contacts denoted as a dot (top), and as percentage distribution intervals (95/75/50/25/5) of contact errors (middle) and bit errors (bottom). Programming errors are largely chip-specific, with 3 chips (#’s 8–10) not displaying any programming errors. **(B)** Heatmaps of programming contact errors for each chip demonstrate that errors cluster on similar regions for each chip. **(C)** There was no significant trend that programming time affected programming error rate, indicating that chip-specific programming errors are independent of the clock rate.

We tested ten μDBS chips for programming fidelity (Figure 5): chips must be programmed properly in order to stimulate properly. We randomized a series of (216 contacts × 3 bits/contact =) 648 bits for each programming trial, to give a diverse range of maximally disordered configurations. The minimal programming duration for a single chip was limited to ∼2.7 s by the maximal clock rate of the Arduino device we used for programming. Since a complete μDBS lead comprises four flat chips, programming an entire lead with this device would take ∼10.8 s in total. Programming times of 2.7–14.3 s at six different clock speeds were tested five times each, for a total of thirty programming sessions per chip. Some chips exhibited no mutation errors in any session, and there was no significant relationship between programming time and error rate (Figure 5C, *p* = 0.97, ANOVA). Thus, chips could likely be programmed in much less than 2.7 s, given appropriately high-clock rate controllers.

Figure 5A reports that three chips (#’s 8–10) did not have any errors regardless of settings, and four others (#’s 4–7) had relatively rare and/or constrained errors. In one of its thirty trials, chip #7 encountered a single deletion error, where one missed bit initiated a cascade effect resulting in many improperly set contacts in that one trial. However, most of the errors were programming mutations, where one bit was toggled inappropriately. Because individual contacts are so small and operate in parallel with the other contacts composing a shared electrode, lone mutation errors would not substantially impact functionality. Across all randomized trials, >95% of errors arose from a mutation toggling one bit from a low to a high state, which indicates possible crosstalk between programming connections. Contact errors are summarized in heat maps on each chip in panel Figure 5B. Most errors on any given chip recurred at similar locations — denoted with yellow-to-red coloring — which may indicate circuit damage that could have occurred during handling.

### Electrode Testing

The programming tests verified that each contact received an appropriate bus line command, but verifying that the contacts successfully link to the intended bus line requires electrical testing of the electrodes. For electrical testing, we submerged μDBS chips in a saline bath and programmed them. In separate experiments, we measured the effective impedance of electrodes built from various numbers of contacts, and assessed the spatial voltage profile generated by two separate electrodes driven by two separate bus lines on the same chip.

**FIGURE 6:**
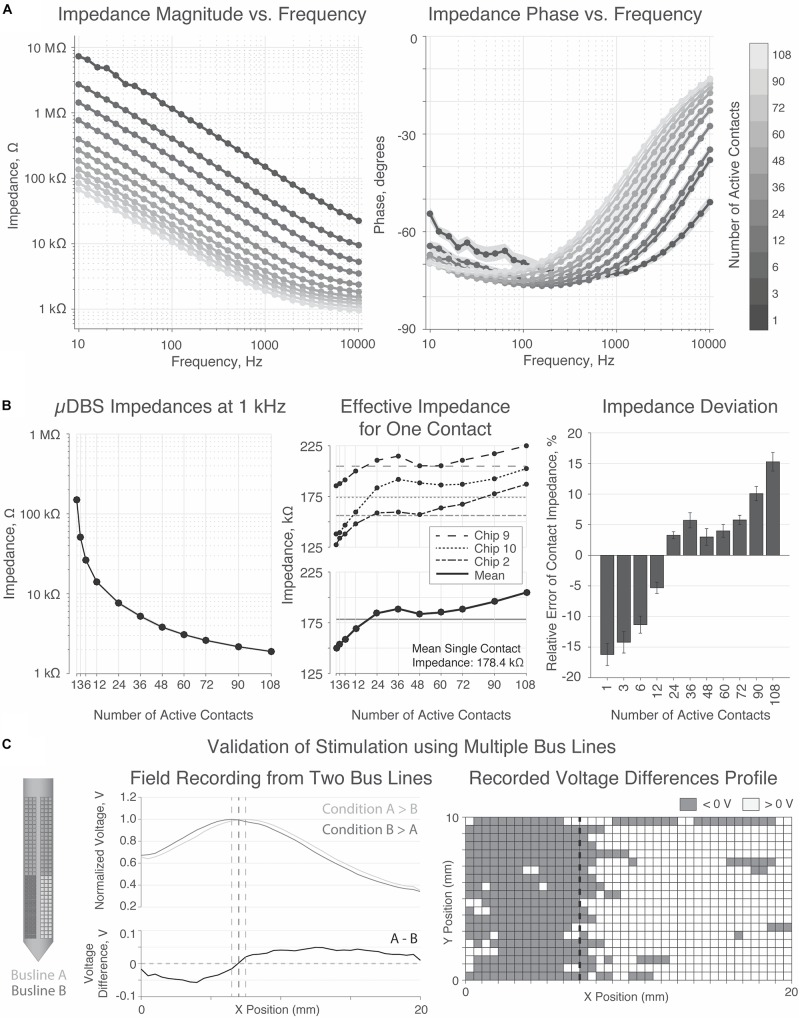
Impedance and bath testing validation. **(A)** Magnitude and phase of impedance for 1 through 108 contacts activated. Impedance decreased at higher frequencies. **(B)** Impedance was inversely proportional to surface area. Average impedance for a single contact was 178.4 kΩ, with a trend toward increasing with the number of active contacts. **(C)** Bath testing demonstrates a directional shift in the normalized voltage field depending on the relative amplitudes of the electrode voltages.

Three chips were submerged into saline solution and connected to a computer for programming and a potentiostat for impedance testing according to Figure 4B. Impedances were recorded on each chip for electrodes programmed to range from 1 to 108 contacts, for a range of frequencies. Each recording was repeated three times, and the resulting impedance magnitude and phase spectra are shown in Figure 6A. Consistent with studies of other electrodes, impedances were higher at lower frequencies due to capacitance at the electrode-tissue interface.

As expected, impedance was inversely proportional to the electrode surface area — i.e., the number of active contacts—supporting that the contacts were properly programmed and linked to the appropriate bus line. Figure 6B summarizes impedance values at 1 kHz, the frequency most commonly used to report impedances of clinical DBS devices. At 1 kHz, a single contact has an impedance of ∼180 kΩ, yielding an effective electrode impedance of ∼180 kΩ divided by the number of constitutive contacts. Thus, an electrode comprising 90 contacts has a surface area of ∼2.0 mm^2^ and an impedance of ∼2.0 kΩ, matching (to within a few percent) the corresponding parameters of clinically approved directional electrodes (Butson et al., 2006; Rebelo et al., 2018).

Finally, the field-testing experiment from Figure 4C was performed on one chip to validate stimulation fields generated from two simultaneously active bus lines (Figure 6C). Two groups of 48 contacts on each half of the chip were tied to one of the two bus lines. In the two conditions tested, either bus line A was greater than bus line B, or vice versa. The voltage probe, traveling in a 20 mm × 10 mm grid in front of the stimulation electrodes, recorded a shift in the peak voltage based on which side of the μDBS lead was tied to the larger amplitude bus line (*p* < 0.00001, two-sample *t*-test). Although our experimental configuration did not allow for a comprehensive mapping of the voltage field, these results demonstrate that the distinct electrodes on opposite sides of a μDBS chip are capable of properly stimulating with separate voltage signals.

### Computational Experiment

**FIGURE 7:**
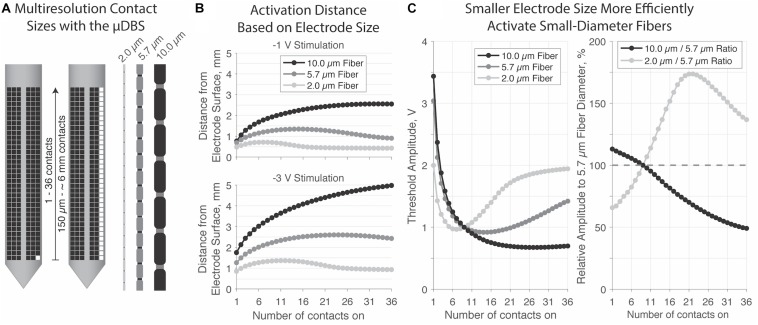
**(A)** Multicompartment axon models were run with diameters of 2.0, 5,7, and 10.0 μm in response to stimulation from 1 to 36 contacts on the μDBS. **(B)** Maximum activation distance (mm) for each fiber size based on electrode size at –1 V (top) and –3 V (bottom). Large-diameter fibers can be activated at greater distances away from the electrode. For smaller diameter fibers, larger electrode sizes reduce activation spread. **(C)** Firing threshold was normalized to –1 V amplitude at 9 contacts on, which is approximately the height of classic DBS contact of 1.5 mm. A multi-resolution device may be useful to target different diameter fibers; as shown in the right panel, smaller contacts activate small diameter fibers at 65.75% efficiency over 5.7 μm fibers, and require about 113.1% additional voltage to activate the same 10.0 μm fibers. Larger contact sizes preferentially activate large diameter fibers, with about 50% lower thresholds relative to 5.7 μm fibers.

Our final experiment demonstrates a possible advantage to a multiresolution device like the μDBS. We modeled individual axons in NEURON responding to voltage fields generated via μDBS electrodes as simulated in SCIRun. Initial electrodes were modeled as 9 vertically stacked contacts, or a 1.47 mm electrode height, to approximate the extent of standard cylindrical DBS electrodes. Modeling axons of three diameters—2.0, 5.7, and 10.0 μm—running parallel to the electrode, we positioned each fiber to its threshold distance at which a −1 V stimulation elicited an action potential. We then varied the number of active contacts within the electrode from 1 to 36, and determined the threshold voltage at which each axon fired (Figure 7).

The distance from the electrode surface at which larger axons can be activated is greater than the activation distance for smaller axons for all electrode sizes. Initially, as the electrode size increases, activation spread increases, however, for smaller diameter fibers the extent of activation reduces as electrode size continues to increase, especially with 2.0 μm fibers. When considering relative activation across different fiber sizes, smaller electrodes preferentially excited smaller axons. In the extreme case of a single-contact electrode (i.e., 150 μm), 2.0 μm fibers were activated at 66 and 58% of the 5.7 and 10.0 μm fiber thresholds, respectively (Figure 7C, right). Conversely, larger electrodes preferentially excited larger axons. In the extreme case of a 36-contact electrode (i.e., ∼6 mm), 10.0 μm fibers were activated at 50 and 36% of the 5.7 and 2.0 μm fiber thresholds, respectively. Thus, the ability to use smaller electrodes may open the therapeutic window by increasing the activation of small, nearby, and likely therapeutic fibers, while decreasing the activation of large, distant, and likely side-effect inducing ones.

## Discussion

This manuscript discusses fabrication and testing of the μDBS device, a novel DBS lead with hundreds of individually controllable contacts. We proposed a novel approach to DBS lead design and assembly using silicon-based wafer technology that incorporates onboard circuitry capable of recruiting electrodes in essentially innumerable shapes, sizes, and configurations. A complete μDBS device is composed of four silicon chips, each with 216 contacts, assembled in a plus-shaped configuration (Figure 1B). The manuscript reports on validation tests on flat chips, of which four are needed for the full μDBS device. However, additional work is required to package the complete device and ensure its longevity in tissue and future tests must be conducted to quantify tissue damage during lead insertion to evaluate safety of this novel lead geometry. Assembly of the full μDBS device was not done in this manuscript, but we have previously shown mechanical stability in the 3D configuration (Willsie and Dorval, 2015b). For the chips tested, silicone was used to encase wirebonds and traces exposed on the PCB in the saline bath, but better packaging is necessary for chronic animal studies. In this manuscript, we have demonstrated feasibility of a novel DBS device design and have highlighted the benefits of leads that can stimulate through multiresolution electrodes.

The inclusion of onboard circuitry enables full control of the hundreds of contacts on the μDBS with a minimal numbers of input wires. Clinically approved devices (Medtronic, Boston Scientific, and Abbott Laboratories) require a separate wire for each contact that must fit in the lead shaft and pass under the skin to a pulse generator in the chest; such a design limits the manufacturing feasibility of increasing the number of contacts on a device. Other silicon-based neural probes, such as those created by NeuroNexus, do not use onboard circuitry and would be similarly limited in the total number of wires capable of being connected to a lead. The incorporation of onboard circuitry, however, adds possible failure modes to the lead design, as demonstrated in our programming testing, in which not all of the ten chips tested could be reliably programmed, with damage to the onboard circuitry being the likely culprit. The programming experiment in Figure 5 demonstrates that errors, which could arise and incorrectly assign contacts to bus lines, are device dependent and cluster together on the chips, however, some chips did not display any programming errors regardless of programming time. Specifically, we found that the transition from 0 bit to 0 bit states was the most common mutation error (95%) during randomized bit programming. It may be possible in the future to reduce these programming errors by reducing the noise in the onboard circuitry with improvements in the integrated circuit design. Finally, inclusion of onboard circuitry increases the energy demands of the lead, and in the current iteration, the μDBS must be powered during programming and stimulation. It may be possible, however, to improve circuit efficiency and explore strategies to reduce energy usage by powering the device only during stimulation pulses and not during the interpulse period.

The impedances reported for individual contacts were around 180 kΩ on average for three chips, but single contact impedances varied on a chip-by-chip basis, which may be the result of slight variations in the post-processing of chips shown in Figure 3. FDA safety standards of charge density would need to be followed during stimulation parameter selection, especially for small electrodes sizes. For small electrodes, resulting volumes of tissue activated would be small since very little charge could be injected into the tissue while staying within safety limits. Alternative processing techniques in future work could decrease the impedance of individual contacts to facilitate stimulation of tissue. These techniques could include the deposition of other metals, such as iridium oxide, with an increased effective contact surface area through its coral-like structure (Negi et al., 2010), or platinum iridium, as used in clinically approved devices. As shown in Table 1, our gold contact sizes were slightly smaller than originally intended, contributing to the higher impedance values, however, compensating for underetching by using larger contact masks in future iterations will decrease the contact impedances.

The novel design of the μDBS does not merely enable smaller electrode contacts, but also offers the ability to combine individual contacts into larger electrodes, even larger than what is clinically available. The total surface area of a stimulation electrode depends on how many contacts are tied to the same bus line, which is demonstrated in Figures 4A,B. Contacts grouped together function as larger electrodes and can mimic the size of clinical contacts, having similar impedances to those recorded in the clinic. The differing impedance levels recorded based on surface area demonstrates that contacts can be recruited appropriately for larger electrodes. Multiresolution stimulation contact sizing affords the μDBS an unprecedented level of flexibility, which could be useful in both research and clinical applications. Multiresolution electrodes can be especially beneficial in the customization of volumes of tissue activated based on patient-specific brain imaging and neural targets. Finally, flexibility in electrode sizes could be useful as more neurological disorders, especially psychiatric disorders with fiber tracts as targets, are being investigated for DBS therapy. Given that it would be impossible to manually choose optimal contact configurations for such a device, we have previously published an optimization algorithm that can identify optimal contact amplitudes and configurations in near real-time based on patient-specific imaging and neural structures (Anderson et al., 2018b).

The large number of electrode configurations and the seven possible voltages states through the seven bus lines on the μDBS allow for highly precise activation field shaping. We tested field shaping through recorded voltage profiles in a saline bath with two groups of contacts tied to one of two active bus lines, with one bus line set to twice the amplitude of the other. When the bus lines were swapped, there was a notable shift in the voltage field, and this demonstrates how the μDBS is able to recruit contacts to separate bus lines simultaneously (Figure 6C). We have previously shown through computational modeling that the μDBS is capable of precise field steering given instances where lead placement error has resulted in suboptimal lead placement off from its target by a few millimeters (Willsie and Dorval, 2015a). Our directional steering work using the μDBS corroborates other studies for different directional lead designs which have shown that smaller, directional contacts are able to activate neural structures while avoiding side-effect-inducing regions (Contarino et al., 2014; Pollo et al., 2014; Steigerwald et al., 2016; Dembek et al., 2017). Additionally, we have found that the use of smaller contacts goes beyond improved field shaping capability: smaller electrodes increase the selectivity of smaller axons compared to larger axons (Figure 7). Mechanistically, we believe that this selectivity is due to the smaller internode spacing of small-diameter axons which can more readily detect the spatially fine changes in the voltage field cased by smaller sized contacts. An increased selectivity for smaller diameter fibers could be used to improve therapeutic DBS since smaller fibers are typically associated with clinical benefit whereas larger fibers are more associated with side effects (Lang et al., 1999; Chaturvedi et al., 2010). For the computational selectivity experiments in this manuscript, we limited our study to vertical neurons to match the orientation of the contacts on the μDBS we studied, but as we previously have shown, different neuron orientations can change the activation profiles (Anderson et al., 2018a).

## Conclusion

The μDBS is a novel DBS device with hundreds of microscale contacts and seven independent voltage states capable of fine control of stimulation fields through multipolar and complex bipolar configurations. This device lays the groundwork for the technology required to increase lead complexity that will allow for more stimulation contacts without the addition of more wires, and may enable the field of DBS technology to move from the initial DBS design that has been used for decades toward directional leads with a much greater number of smaller contacts. This device is the first of its kind that features multiresolution electrodes, which can be used to morph stimulation fields to the often irregular size and shape of neural targets and can offer stimulation flexibility in novel applications of DBS where stimulation targets are still being explored. Finally, we present novel evidence that smaller, directional contacts may be even more advantageous for stimulation therapy than currently thought: not only is there greater field shaping flexibility with directional contacts, but smaller contacts also improve targeting of smaller diameter fibers, which may lead to increases in the therapeutic window.

## Data Availability Statement

The datasets generated for this study are available on request to the corresponding author.

## Author Contributions

AD conceived of the device design and project concept. DA executed the VLSI design, post-processed the devices, tested the devices, analyzed the data, ran the computational model, and wrote the manuscript. NL assisted in the VLSI design of the device and post-processing of the devices. CA assisted in post-processing and testing of the devices and analysis of the data. RS developed the packaging procedure. CB provided instruction on computational modeling. BB provided training and troubleshooting of the post-processing steps of the device in the Utah Nanofab cleanroom.

## Conflict of Interest

CB has served as a consulting for NeuroPace, Advanced Bionics, Boston Scientific, Intelect Medical, Abbott (St. Jude Medical), and Functional Neuromodulation. CB is also a shareholder of Intelect Medical and is an inventor of several patents related to neuromodulation therapy. The remaining authors declare that the research was conducted in the absence of any commercial or financial relationships that could be construed as a potential conflict of interest.
